# Evaluation of Two Strategies for Community-Based Safety Monitoring during Seasonal Malaria Chemoprevention Campaigns in Senegal, Compared with the National Spontaneous Reporting System

**DOI:** 10.1007/s40290-018-0232-z

**Published:** 2018-06-01

**Authors:** Jean-Louis A. Ndiaye, Ibrahima Diallo, Youssoupha NDiaye, Ekoue Kouevidjin, Ibrahima Aw, Fassiatou Tairou, Tidiane Ndoye, Christine M. Halleux, Isaac Manga, Mbaye Niang Dieme, Medoune Ndiop, Babacar Faye, Piero Olliaro, Corinne S. Merle, Oumar Gaye, Paul Milligan

**Affiliations:** 1 Department of Parasitology, Cheikh Anta Diop University, Thies University, Dakar, Senegal; 2 National Malaria Control Programme, Ministry of Health and Social Affairs, Dakar, Senegal; 3 Sedhiou Medical Region, Ministry of Health and Social Affairs, Sedhiou, Senegal; 4 Department of Parasitology, Cheikh Anta Diop University, Dakar, Senegal; 5 Department of Social Sciences, Cheikh Anta Diop University, Dakar, Senegal; 6 The Special Programme for Research and Training in Tropical Diseases, World Health Organization, 1121 Geneva 27, Switzerland; 7 Department of Infectious Disease Epidemiology, London School of Hygiene and Tropical Medicine, London, WC1E 7HT UK

## Abstract

**Background:**

Seasonal malaria chemoprevention (SMC) using sulfadoxine–pyrimethamine plus amodiaquine has been introduced in 12 African countries. Additional strategies for safety monitoring are needed to supplement national systems of spontaneous reporting that are known to under represent the incidence of adverse reactions.

**Objectives:**

This study aimed to determine if adverse event (AE) reporting could be improved using a smartphone application provided to village health workers, or by active follow-up using a symptom card provided to caregivers.

**Methods:**

Two strategies to improve reporting of AEs during SMC campaigns were evaluated, in comparison with the national system of spontaneous reporting, in 11 health post areas in Senegal. In each health post, an average of approximately 4000 children under 10 years of age received SMC treatment each month for 3 months during the 2015 malaria transmission season—a total of 134,000 treatments. In three health posts (serving approximately 14,000 children), caregivers were encouraged to report any adverse reactions to the nurse at the health post or to a community health worker (CHW) in their village, who had been trained to use a smartphone application to report the event (enhanced spontaneous reporting). In two health posts (approximately 10,000 children), active follow-up of children at home was organized after each SMC campaign to ask about AEs that caregivers had been asked to record on a symptom card (active surveillance). Six health posts (approximately 23,000 children) followed the national system of spontaneous reporting using the national reporting (yellow) form. Each AE report was assessed by a panel to determine likely association with SMC drugs.

**Results:**

The incidence of reported AEs was 2.4, 30.6, and 21.6 per 1000 children treated per month, using the national system, enhanced spontaneous reporting, and active surveillance, respectively. The most commonly reported symptoms were vomiting, fever, and abdominal pain. The incidence of vomiting, known to be caused by amodiaquine, was similar using both innovative methods (10/1000 in the first month, decreasing to 2.5/1000 in the third month). Despite increased surveillance, no serious adverse drug reactions were detected.

**Conclusion:**

Training CHWs in each village and health facility staff to report AEs using a mobile phone application led to much higher reporting rates than through the national system. This approach is feasible and acceptable, and could be further improved by strengthening laboratory investigation and the collection of control data immediately prior to SMC campaigns.

**Electronic supplementary material:**

The online version of this article (10.1007/s40290-018-0232-z) contains supplementary material, which is available to authorized users.

## Key Points

**Table:** 

Seasonal malaria chemoprevention (SMC) is now widely used to prevent malaria in children in West and Central Africa. Good safety monitoring is essential to ensure SMC programs remain effective.
Training community health workers (CHWs) to recognize and report adverse events (AEs) improved detection of adverse drug reactions in this study in Southern Senegal.
Training CHWs and health facility staff to report using a mobile phone application enhanced safety reporting and improved timeliness of notifications during SMC campaigns.
No serious AEs were detected despite enhanced surveillance.

## Introduction

Malaria remains a major public health concern in the world, particularly in sub-Saharan Africa. The World Health Organization (WHO) estimates 216 million malaria cases and 445,000 malaria deaths occurred in 2016 [1], with the vast majority of malaria deaths occurring in children in sub-Saharan Africa caused by *Plasmodium falciparum*. Since 2012, the WHO has recommended seasonal malaria chemoprevention (SMC), consisting of the monthly administration of a full course of treatment with sulfadoxine–pyrimethamine (SP) and amodiaquine (AQ), during the transmission season to prevent malaria. Although SMC is recommended for children aged 3–59 months [2], in Senegal (as in many other areas where SMC is used) there is a substantial burden of severe malaria illness in older children. The Senegalese Ministry of Health made the decision to provide SMC for children up to 10 years of age when SMC was first introduced. After a pilot implementation in 2013, SMC was implemented in four regions of the country where malaria transmission was most intense (Kedougou, Kolda, Tambacounda and Sedhiou), in a population of approximately 600,000 children. From 2013 to 2017, approximately 8 million treatments were administered. A total of seven serious adverse events (SAEs) related to SMC have been reported in Senegal since the introduction of SMC, up to 2017: a case of Stevens–Johnson syndrome and a case of toxic epidermal necrolysis, both in 2014; a case of extrapyramidal syndrome and two cases of anaphylactic reactions in 2015; and one case of Stevens–Johnson syndrome and one anaphylactic reaction in 2016. No SAEs were reported in 2017. Although pharmacovigilance during SMC campaigns was strengthened through training of health staff to recognize adverse reactions to SMC drugs, there is concern that adverse events (AEs) are being underreported. Known adverse reactions to SP and AQ have been reviewed by Phillips-Howard and Bjorkman [3] and, more recently, NDiaye et al. [4]. AQ is associated with vomiting, extrapyramidal reactions, liver toxicity, and agranulocytosis, while SP can cause liver toxicity and severe cutaneous reactions, including Stevens–Johnson syndrome and toxic epidermal necrolysis, which are rare but life-threatening medical emergencies.

Pharmacovigilance systems based on spontaneous reporting are relatively simple and inexpensive to establish but often suffer from poor quality of reporting, as well as underreporting [5]. In addition, it is difficult to estimate incidence rates of AEs through a spontaneous reporting system, generally because of lack of a denominator (number of people exposed). Additional methods are needed to determine the safety profile of new medicines, as well as medicines put to new uses, in public health programs [6]. This is especially important in the framework of mass administration of preventive treatment to children, delivered by community workers with limited medical training. SAEs to SMC drugs appear to be uncommon, but, especially in the initial phases of SMC programs, there is a need to supplement the national system of spontaneous reporting with additional methods of surveillance.

The aim of this study was to evaluate whether AE reporting could be improved using one of two approaches: (1) reporting using a smartphone application when patients presented at the clinic or to a village health worker; and (2) active follow-up of children at home to ask about AEs and collect information caregivers were asked to record on a symptom card, compared with the current national system for spontaneous reporting alone. Feasibility and acceptability of the implementation of the strategies were also assessed.

## Methods

### Study Population

This study was conducted in the health districts of Kolda and Sedhiou in the south of Senegal (Electronic Supplementary Fig. S1) during the SMC campaign of 2015 (August, September, and October). The population is primarily rural. In 2016, the under-5 mortality rate was estimated to be 100 per 1000 children, and the literacy rate was 55% in men and 41% in women [7].

Malaria is a major cause of severe illness in children. Community case management for malaria is provided in the more remote villages by a community health worker (*Distributeur de Soins à Domicile* [DSDOM]) working from their home or at a *case de santé* (health hut). SMC targeted children aged 3 months to 10 years and is delivered door-to-door by the DSDOM and (in villages that do not have a resident health worker) by relais communautaires (community volunteers). In this report, DSDOM and relais communautaires are collectively referred to as community health workers (CHWs). SMC delivery is coordinated by the health post from where CHWs collect drugs each day, with SMC distribution following general WHO recommendations [8]. One dose of SP and the first dose of AQ were administered by the CHW on the first day, and the remaining two doses of AQ were left with the caregiver to administer over the next days. Children who are unwell are referred without treatment, however they may then receive SMC at the health post. CHWs are trained to exclude children with a history of allergy to SMC drugs and any child who had received SP, AQ, or a sulfa-containing antibiotic in the previous month. Treatments administered are recorded on tally sheets that are collated to give monthly totals.

### Study Design

Eleven health posts in SMC implementation areas were selected and assigned to monitor SMC safety using one of three methods: safety monitoring employing the national system of spontaneous reporting using the national reporting form (referred to as the national system), completed by nurses or physicians at health facilities; reporting using mobile phones (enhanced spontaneous reporting), completed by nurses at health posts and by CHWs; and active follow-up of children at home after each SMC campaign to ask about AEs and collect information that caregivers had been asked to record on a symptom card (active surveillance). Enhanced spontaneous reporting with mobile phones was implemented in two health posts in Kolda, and active surveillance was implemented in three health posts in Sedhiou. For comparison, three health posts in each district, where the staff were trained to report events using the national reporting form, acted as controls.

### Sample Size, Allocation, and Description of Surveillance Methods

In each district, two groups of health posts were selected, with a total of approximately 10,000 children per group. The two groups were then randomly allocated within each district to either control or enhanced or active surveillance. The sample size of 10,000 children permits event rates above 0.3/1000 children to be ruled out if zero events are observed, and is sufficient to give 99% probability of detecting at least one event if the rate is 1/2000 or more. As SMC was administered to each child up to three times, the power for detecting events per 1000 treatments is greater. Two health posts in Sedhiou (Diendé and Dembo Coly, including 53 villages) were assigned to enhanced spontaneous reporting, and three health posts in Kolda (Bagadadji, Dabo and Sikilo Ouest, including 106 villages) were assigned to active surveillance. Six health posts, three in each district, assigned to the national system, acted as controls (Table 1). In the area assigned to active surveillance, consent to participate in the study was sought when CHWs visited to administer the first monthly SMC treatment.

**Table 1:** Number of staff trained for pharmacovigilance in each health facility, size of the catchment population, number of children eligible for SMC, and number of children who received SMC treatment each month

District	Health post	Method used for PV	No. of health staff trained for PV: nurses (CHWs)	Total population in 2015	SMC target population	No. of children who received SMC treatment		
						September	October	November
Sedhiou	Diende	Enhanced spontaneous reporting	1 (9)	21,958	6587	4787	4849	5134
	Dembo Coly	Enhanced spontaneous reporting	1 (9)	25,486	7646	7545	7574	7569
	Djibabouya	National system	1 (10)	12,228	3668	2862	3008	3067
	Bambaly	National system	1 (10)	17,162	5148	3851	4100	4509
	Djiredji	National system	1 (10)	11,337	3401	3956	4004	3976
Kolda	Bagadadji	Active surveillance	1 (6)	14,862	4459	4393	4454	4576
	Dabo	Active surveillance	1 (6)	9705	2911	3057	3058	3065
	Sikilo Ouest	Active surveillance	1 (10)	8011	2403	2732	2994	3123
	Sikolo Est	National system	1 (10)	9412	2824	2794	2654	2675
	Dioulacolon	National system	1 (10)	16,619	4986	4997	4974	5121
	Guiro Yero Boucar	National system	1 (10)	9560	2868	2824	2925	2854

*PV* pharmacovigilance, *CHWs* community health workers, *SMC* seasonal malaria chemoprevention

### Enhanced Spontaneous Reporting

At the time of each monthly SMC distribution, caregivers were encouraged to contact the nearest CHW (DSDOM) or the health post if the child was unwell after SMC administration. The nurses and the CHWs first entered the patient details in the consultation register and treated or referred the patient as appropriate. Then, in the case of children over 3 months and under 10 years of age who had received SMC and had any illness, the nurses and CHWs entered the name, age and sex of the child, all medicines received in the last month and the date of administration, and the date of onset of symptoms and a description of the symptoms, into a smartphone application which then uploaded the data over the internet to a server in the project office in Dakar. The application was designed using Survey CTO (Dobility, Inc., Wilmington, DE, USA), a platform based on Open Data Kit (ODK), an open source set of programming tools for data capture. The date of the report was generated automatically. The phones were Samsung Duo, costing CFA 30,000 each (US $51 at 2015 exchange rates), provided with SIM cards and internet credit to cover the costs of data uploading. The project team sent an SMS to each health worker each day to feed back to them the number of reports received from them the previous day and the total number from the start of the study. The team contacted health workers who had not submitted any report the previous day to confirm there were no events. A database of all events reported was updated daily and shared with the project team. If the nurse suspected the illness was an adverse drug reaction (ADR), a national reporting form (yellow form) was completed.

### Active Surveillance

At the time of each monthly SMC administration, CHWs delivering SMC gave caregivers a card for each child, which showed images illustrating fever, vomiting, rash, and pain. The caregiver was asked to tick the card if the child had one of these symptoms or any other symptoms at any time after SMC administration. The same CHWs went back to each household after the end of each monthly campaign, between 6 and 12 days after the first day of the SMC cycle, to ask caregivers about any AEs in children who had received SMC. CHWs asked about additional details, the date symptoms started, and, if the child no longer had symptoms, the date symptoms stopped, which the CHW then recorded on the card. The cards were collected and taken to the health post, where the nurse reviewed them before entering the information into a Microsoft Excel database. If the child was still unwell when the CHW visited, the child was referred to the nurse at the health post, who completed a national reporting (yellow) form.

### National System

The nurse at each health post was trained to complete a national reporting form (yellow form) for any child presenting to the health post with suspected AEs, as per the national pharmacovigilance guidelines [6]. During SMC campaigns, yellow forms from all health posts were collected at the district health center. District teams could then enter the forms into a Microsoft Excel database that was forwarded to the regional health team, or the forms were sent to the regional team where they were entered. A final database was then sent to the *Centre Anti-poison* and the National Malaria Control Programme each month.

### Training

An information sheet describing the symptoms of the known AEs and highlighting vomiting, skin rash, and signs of jaundice was prepared and used to train health staff. In all health posts, training for SMC delivery included health facility staff and CHWs, and covered key messages about pharmacovigilance, the symptoms of the known ADRs to SMC drugs, and how to report events using the national form. In addition, for staff in health posts using the smartphones, training was held in each district over 2 days to explain how to report using the smartphone application. Training included practical sessions on AE reporting, and emphasized the importance of checking information before uploading, and the responsibilities of the nurses and CHWs in pharmacovigilance.

### Sensitization and Study Preparation

Meetings were held with regional and district medical officers to emphasize the importance of ensuring that laboratories had reagents for liver function tests and hematology. In each district, a hospital pharmacist was nominated to be the pharmacovigilance coordinator, and a study project manager was appointed. In all health posts, communities were informed about the SMC campaign, including reminders to bring the child to a health worker if the child was sick after taking SMC medicine. In addition, in health posts with enhanced passive or active surveillance, additional community sensitization was organized by the health post nurse.

### Data Management and Statistical Analysis

Incidence rates were calculated per 1000 child months, using the estimated number of children who received SMC each month as the denominator. Rate ratios were used to compare rates between surveillance methods, age groups, and calendar months, estimated using Poisson regression with a robust standard error to account for clustering within health posts and with stratification by district. The mean number of symptoms reported per patient was compared with Poisson regression. To assess whether particular symptoms tended to be reported together, odds ratios for associations between symptom pairs were compared between groups, using a test of homogeneity, for the four most commonly reported symptoms. Data were analyzed using Stata 13 (StataCorp LLC, College Station, TX, USA).

### Causality Assessment

Health workers were asked to report any illness as AEs not necessarily having a causal relationship with medical treatment. The Centre Antipoison of Senegal and the pharmacovigilance Technical Committee analyzed the reports and assessed the severity and association with medicine intake following the WHO method [9]. All case reports were submitted to the international drug monitoring database through Vigiflow.

### Ethics

The research protocol and documents given to participants were submitted to the National Ethics Committee of Senegal and approval obtained prior to the start of the study. Administrative authorization was also given by the Ministry of Health. Community consent was obtained for the phone-reporting pharmacovigilance prior to the beginning of the study. For children in the active surveillance group, signed consent was sought from a parent or guardian after explaining the aims and procedures involved. An advisory committee was set up by the National Malaria Control Programme at central level to provide guidance on the management of any severe AEs during SMC campaigns.

## Results

A total of 1983 AEs were reported over the 3 months of surveillance, out of a total of 134,061 monthly treatments. Of these, 158 were reported through the national system (a rate of 2.4/1000 children treated/month), 1145 (31/1000 children/month) were reported through the enhanced spontaneous reporting system using CHWs and mobile phones, and 680 (22/1000 children/month) were reported through active surveillance. All patients reported having taken SMC drugs, and 154/1983 (7.8%) had taken one or more other medications in addition to SMC drugs, comprising 53 who had taken camphorated tincture of opium (paregoric, a diarrhea treatment including 0.4 mg/mL morphine), 49 who had taken metopimazine (an antiemetic), 43 who had taken paracetamol, 24 who had taken zinc, 7 who had taken phloroglucinol (a treatment for abdominal pain), 2 who had taken amoxicillin, 2 who had taken mequitazine (an antihistamine), 2 who had taken metronidazole (an antibiotic used to treat protozoal infection), 1 who had taken chloroquine, and 1 who had taken traditional medicine. None of the events reported were considered serious.

### Enhanced Spontaneous Reporting

**Fig. 1:**
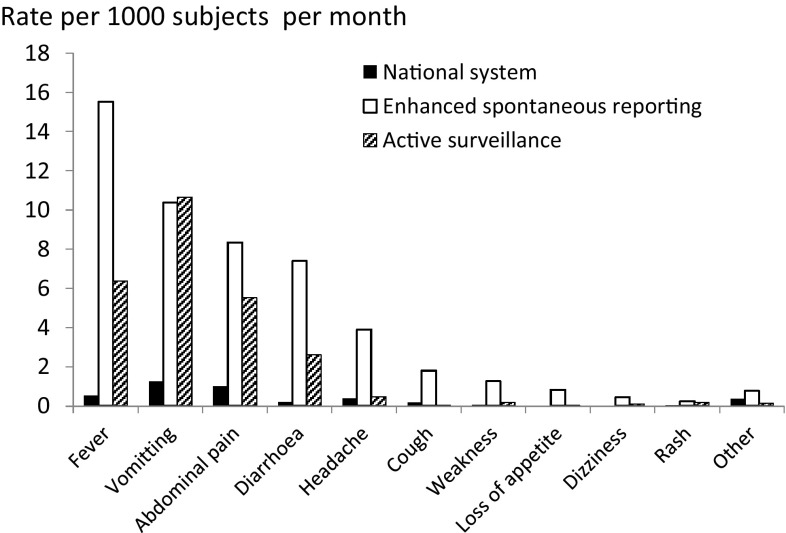
Average incidence of each symptom, as reported through each method of surveillance

**Fig. 2:**
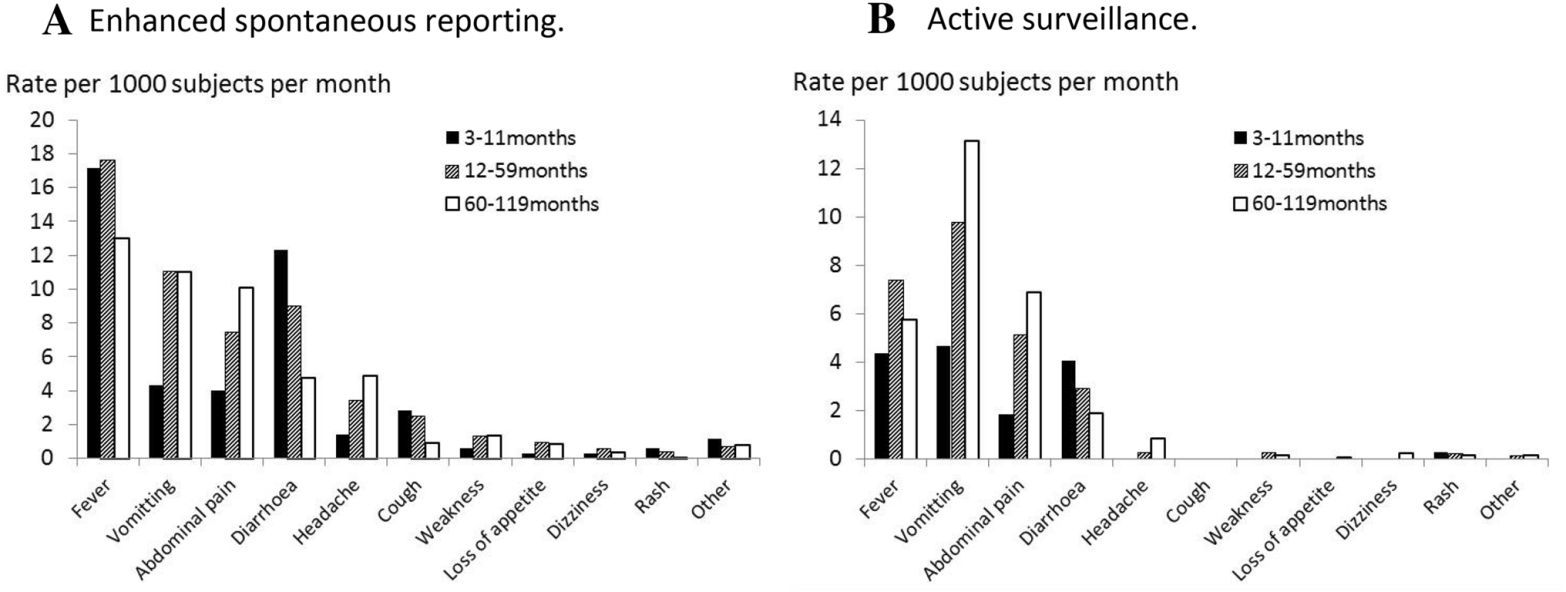
Incidence of adverse events, by age group, for each surveillance method. **a** Enhanced spontaneous reporting; **b** active surveillance

Overall, 1145 events were reported over 3 months, a rate of 30.6 [95% confidence interval (CI) 28.8–32.4] per 1000 children treated per month, compared with a rate of 1.65 (95% CI 1.27–2.15) per 1000 per month in health posts using the national system (Table 2). A total of 927 events (81% of the total) occurred within 10 days of the start of the SMC cycle. The incidence of AEs decreased in each successive month, from 30.1/1000 in September, to 25.2 in October and 10.1 in November. The incidence was slightly lower in infants than in older children (Table 4). The most commonly reported symptoms were fever, vomiting, and abdominal pain (Fig. 1). Among the older children (5–10 years of age), the most common symptoms were, in descending order, fever, vomiting, abdominal pain, headache, and diarrhea (Fig. 2), with a similar pattern being observed in the 12–59 months age group. Among children aged 3–11 months, the most common symptoms were fever and diarrhea. Tiredness, cough, loss of appetite, dizziness, and pruritus were less commonly reported, and other symptoms (Electronic Supplementary Table S4) together accounted for < 1% of reported symptoms. No AE was considered serious. The average number of symptoms reported per event was 1.67, with 50% of patients reporting more than one symptom. The distribution of the number of symptoms per event is shown in electronic supplementary Table S2. Symptoms varied by age group (Fig. 2). Vomiting was reported at a rate of 10.7/1000/month (Table 3), and was most commonly associated with diarrhea or fever (electronic supplementary Table S3). When the reports were assessed for their relationship with SMC drugs, 27% of AEs were considered probably related to SMC, 36% were possibly related, 1.2% were unlikely to be related, and causality could not be assessed for the remaining 36%.

**Table 2:** Incidence of adverse events (reports with one or more symptoms) following SMC distribution, using three methods of surveillance

	No. of events	No. of treatments	Incidence rate/1000 (95% CI)	Incidence rate ratio (95% CI)^a^
*Kolda*				
National system	103	31,818	3.2 (2.7–3.9)	Reference
Active surveillance	680	31,452	21.6 (20.1–23.3)	6.7 (1.3–33.9)
*Sedhiou*				
National system	55	33,333	1.65 (1.3–2.1)	Reference
Enhanced spontaneous reporting	1145	37,458	30.6 (28.8–32.4)	18.5 (8.65–39.7)

*SMC* seasonal malaria chemoprevention, *CI* confidence interval

^a^Incidence rate ratios comparing the surveillance methods were estimated using Poisson regression, with the estimated number of SMC treatments as offset, and using robust standard errors to account for clustering within health post

**Table 3:** Incidence of vomiting following SMC distribution, using three methods of surveillance

	No. of events	No. of treatments	Incidence rate/1000 (95% CI)	Incidence rate ratio (95% CI)
*Kolda*				
National system	47	31,818	1.5 (1.1–2.0)	Reference
Active surveillance	335	31,452	10.7 (9.6–11.9)	7.2 (1.8–29.1)
*Sedhiou*				
National system	34	33,333	1.0 (0.73–1.4)	Reference
Enhanced spontaneous reporting	389	37,458	10.4 (9.4–11.5)	10.2 (5.8–18.0)

*SMC* seasonal malaria chemoprevention, *CI* confidence interval

**Table 4:** Incidence of adverse events (reports with one or more symptoms) following SMC distribution, using three methods of surveillance, by age group and month of administration

	No. of events	No. of treatments	Incidence/1000	Incidence rate ratio (95% CI)
*Kolda*				
*Active surveillance*				
Age group (months)				
3–11	39	3212	12.1	0.56 (0.44–0.70)
12–59	324	15,052	21.5	Reference
60–120	317	13,188	24.0	1.12 (1.10–1.15)
Month				
September	306	10,182	30.1	Reference
October	265	10,506	25.2	0.84 (0.35–2.03)
November	109	10,764	10.1	0.33 (0.09–1.26)
*Kolda*				
*National system*				
Age group (months)				
3–11	4	2842	1.4	0.45 (0.32–0.62)
12–59	48	15,255	3.1	Reference
60–120	51	13,721	3.7	1.16 (0.60–2.24)
Month				
September	77	10,615	7.3	Reference
October	19	10,553	1.8	0.25 (0.09–0.68)
November	7	10,650	0.7	0.09 (0.03–0.25)
*Sedhiou*				
*Enhanced spontaneous reporting*				
Age group (months)				
3–11	114	3491	32.7	1.00 (0.73–1.38)
12–59	557	17,142	32.5	Reference
60–120	474	16,825	28.2	0.85 (0.77–0.94)
Month				
September	570	12,332	46.2	Reference
October	406	12,423	32.7	0.71 (0.38–1.30)
November	169	12,703	13.3	0.29 (0.24–0.35)
*Sedhiou*				
*National system*				
Age group (months)				
3–11	8	2331	3.4	2.57 (1.89–3.48)
12–59	22	16,170	1.4	Reference
60–120	25	14,832	1.7	1.21 (0.46–3.19)
Month				
September	31	10,669	2.9	Reference
October	11	11,112	1.0	0.34 (0.24–0.48)
November	13	11,552	1.1	0.38 (0.24–0.62)

*SMC* seasonal malaria chemoprevention, *CI* confidence interval

In health posts in the same district that used the national system of reporting, there were 55 events, a rate of 1.65/1000/month. Rates similarly declined with each successive month. No AE was considered serious. When reports were assessed for their association with SMC drugs, 11% were considered probably related to SMC, 81% possibly related, 1.2% unlikely to be related, and 6.3% could not be classified.

Enhanced reporting involving CHWs and using mobile phones increased reporting 18-fold (rate ratio 18.5, 95% CI 8.65–39.7), and the incidence of episodes of vomiting by 10-fold (rate ratio 10.2, 95% CI 5.8–18.0).

### Active Surveillance

Overall, 680 events were reported over 3 months, corresponding to a rate of 21.6 (95% CI 20.1–23.3) per 1000 children treated per month, compared with a rate of 3.24 (95% CI 2.67–3.93) per 1000 per month in health posts using the national system. The incidence of AEs decreased in each successive month, from 30.1/1000 in September, to 25.2 in October and 10.1 in November. The incidence rate increased with increasing age, from 12.1/1000/month in infants, to 21.5 in children 12–59 months of age, and 24.0 in older children (Tables 4 and 5). The average number of symptoms reported per event was 1.2, with only one symptom reported in the majority (79%) of events. Among the older children (5–10 years of age), the most common symptoms were, in descending order, vomiting, abdominal pain, fever, diarrhea, and headache. Vomiting was reported at a rate of 10.7/1000/month. Other symptoms included itching, lethargy, cough, loss of appetite, and dizziness, but these were uncommon. No AE was considered serious. When reports were assessed for their association with SMC drugs, 8.6% were considered probably related to SMC, 72% possibly related, 0.24% were unlikely, and 19% could not be classified.

**Table 5:** Incidence of vomiting following SMC distribution, using three methods of surveillance, by age group and month of administration

	No. of events	No. of treatments	Rate/1000 (95% CI)	Rate ratio (95% CI)
*Kolda*				
*Active surveillance*				
Age group (months)				
3–11	15	3212	4.67 (2.82–7.75)	0.47 (0.23–0.95)
12–59	147	15,052	9.77 (8.31–11.48)	Reference
60–120	173	13,188	13.12 (11.30–15.23)	1.36 (1.10–1.67)
Month				
September	171	10,182	16.79 (14.46–19.51)	Reference
October	117	10,506	11.14 (9.29–13.35)	0.66 (0.32–1.37)
November	47	10,764	4.37 (3.28–5.81)	0.26 (0.07–0.99)
*Kolda*				
*National system*				
Age group (months)				
3–11	1	2842	0.35 (0.05–2.5)	0.28 (0.02,3.31)
12–59	19	15,255	1.2 (0.79–1.95)	Reference
60–120	27	13,721	2.0 (1.3–2.9)	1.54 (0.51–4.66)
Month				
September	41	10,615	3.9 (2.8–5.2)	Reference
October	5	10,553	0.47 (0.20–1.14)	0.12 (0.02–0.68)
November	1	10,650	0.09 (0.01–0.67)	0.02 (0.00–0.13)
*Sedhiou*				
*Enhanced spontaneous reporting*				
Age group (months)				
3–11	15	3491	4.30 (2.59–7.13)	0.39 (0.21–0.70)
12–59	189	17,142	11.03 (9.56–12.72)	Reference
60–120	185	16,825	11.00 (9.52–12.70)	0.98 (0.98–0.98)
Month				
September	207	12,332	16.79 (14.65–19.24)	Reference
October	123	12,423	9.90 (8.30–11.82)	0.59 (0.42–0.82)
November	59	12,703	4.65 (3.60–6.00)	0.28 (0.26–0.29)
*Sedhiou*				
*National system*				
Age group (months)				
3–11	4	2331	1.72 (0.64–4.57)	2.82 (1.10–7.28)
12–59	10	16,170	0.62 (0.33–1.15)	Reference
60–120	20	14,832	1.35 (0.87–2.09)	2.13 (0.89–5.10)
Month				
September	20	10,669	1.88 (1.21–2.91)	Reference
October	6	11,112	0.54 (0.24–1.20)	0.29 (0.11–0.77)
November	8	11,552	0.69 (0.35–1.39)	0.37 (0.15–0.94)

*SMC* seasonal malaria chemoprevention, *CI* confidence interval

In health posts in the same district which used the national system of reporting, 103 events (3.24/1000/month) were reported. No AE was considered serious. Twenty percent of these reports were considered probably related to SMC, 70% were possibly related, while the remaining 10% could not be classified. Active surveillance increased reporting almost sevenfold (rate ratio 6.7, 95% CI 1.3–33.9) and reporting of episodes of vomiting by sevenfold (rate ratio 7.2, 95% CI 1.8–29.1).

## Discussion

Although efforts have been made to strengthen national pharmacovigilance capacity during SMC programs [10], it is recognized that national systems based on spontaneous reporting tend to underreport events. There might be multiple reasons for this: (1) events may not be recognized by the health worker as potentially related to administration of the medicine; (2) health staff may be too busy to report the event, or may not know how to report; or (3) the patient may seek care outside the formal health system. Mild and moderate events may be particularly underrepresented as events may not be considered severe enough by a patient to warrant consulting a health worker, or not considered by health workers to be important enough to report, but these could become a concern (jeopardizing acceptability or adherence to treatment) if common. A further problem is that reports are slow to reach national pharmacovigilance and malaria program coordinators, therefore investigation (and the putting in place of any mitigating actions) may be considerably delayed. More intensive monitoring is therefore required, especially in the early phases of a new public health program, to establish its safety [6].

In October 2014, a workshop was organized by the Special Programme for Research and Training in Tropical Diseases (TDR) and the London School of Hygiene & Tropical Medicine (LSHTM) to bring together coordinators of national malaria control programs and national pharmacovigilance centers from countries that had introduced SMC, or planned to in the near future, to discuss with pharmacovigilance experts approaches that could be used to improve safety monitoring of SMC programs. The workshop highlighted the need for operational research into innovative practical methods that could simplify and expedite reporting and improve promptness of notification of events to the national malaria program and the pharmacovigilance center [11]. This led to planning of the present study that evaluated two approaches to community-based pharmacovigilance involving CHWs; one based on passive, spontaneous reporting in which CHWs and health facility staff were trained to report AEs using a mobile phone application, and the other approach involving active follow-up by CHWs after each SMC campaign to ask about AEs that caregivers had been asked to record on a symptom card. Both methods involved sensitization of the community to the importance of reporting suspected AEs, and a central supervision team to process, share, and analyze reports and to provide feedback to reporters.

The incidence rate of reported events was higher using community-based spontaneous reporting—31/1000 children/month, compared with 22/1000 children/month using active surveillance. These rates compare with rates of 1.7–3.2 through the national system in the same districts.

The greater incidence in the enhanced spontaneous reporting arm of the study reflects the longer period of time over which events were observed. In the active surveillance arm of the study, participants were asked about events occurring up to 10 days prior to the visit, whereas any event during the month could be reported in the enhanced spontaneous reporting arm.

When specific symptoms were considered, the incidence rate of vomiting, which, it is known, can be caused by AQ, was seen to be similar in the active and enhanced spontaneous reporting arms of the study in infants and young children, and slightly more common among older children in the active arm of the study, while fever and diarrhea were more commonly reported in the spontaneous reporting arm of the study. It was hypothesized before the study that mild side effects would not be reported spontaneously and active surveillance would be necessary to determine the true burden. The fact that the incidence of vomiting was similar using both methods suggests this is not the case when good access is provided through CHWs based in the village. In the enhanced spontaneous reporting arm of the study, 16 CHWs were based in the community, to whom patients could report, in addition to the two health post nurses. It is likely that this improved access for patients, and the provision of training for these staff to recognize AEs and when and how to report, may have contributed to the high reporting rate, as has been found in other studies [12].

None of the reported AEs was classified as serious. As the number of children who received SMC in the intensified surveillance area was at least 23,000, the upper 95% confidence limit for the rate of severe AEs per child was 1 in 7800 (i.e. on the basis of this study we can rule out rates greater than this).

A limitation of this study is the lack of suitable controls to establish the rate of symptoms in children who did not receive SMC. Data could have been collected prior to the start of the SMC campaigns, although some confounding would remain, as, in this population, morbidity is seasonal, with the rainy season being associated with the transmission of infections and with malnutrition. In this study, in the enhanced and active surveillance areas, health workers were encouraged to report all events. When reports were assessed for evidence of a relationship with SMC drugs, over one-third could not be reliably classified. Therefore, the overall rates are likely to overestimate the true rates of AEs to SMC drugs, but the rates (or rather the upper confidence limits on the rates) can be interpreted as representing an upper bound for the true rate of drug-related symptoms. Furthermore, there may have been reporting bias, both by caregivers who may have been less likely to report symptoms not listed on the symptom card, and by CHWs, whose training emphasized the known side effects of SMC drugs. A further limitation of the study (in common with routine pharmacovigilance) is that although steps were taken to improve laboratory capacity for routine investigation, it was not possible to routinely perform hematological analysis or liver function tests, and therefore it is possible that cases of clinically silent neutropenia (which can be associated with AQ) and hepatitis (which can be associated with both AQ and SP) may have gone undetected. Severe cutaneous reactions, known to be associated with SP, and extrapyramidal syndrome, known to be caused by AQ, are severe and unmistakable, and the fact that no cases of these syndromes were seen suggests it is unlikely that any such events occurred during the study period in the populations under enhanced or active surveillance.

The most commonly reported symptoms were gastrointestinal disorders (nausea/vomiting, abdominal pain, diarrhea), fever, headache, tiredness, cough, loss of appetite, dizziness and pruritus. These symptoms were similar to those reported in other studies of antimalarials in children in the Central African Republic [13], Burkina Faso [14], and Senegal [4], and in school children in Uganda [15]. Fever was more commonly reported in our study compared with other studies on SP + AQ, but this may reflect that the definition of fever includes a reported history of fever, and that the study took place over an extended period during the rainy season, when febrile illnesses are common.

The incidence of AEs decreased in successive rounds of SMC, a finding that is consistent with the results of others studies [4, 14]. The fact that this effect was observed in active and passive surveillance areas suggests there may have been gradual tolerization to the effects of AQ; mothers could also become less likely to report mild symptoms over time.

For reports submitted by mobile phone, the average delay from case presentation to the notification reaching the central office was 24 h. In a study using SMS to monitor AEs after immunization in Australia, most patients who received an SMS query about adverse vaccine reactions sent a response within 2 h of receiving the query [16]. A study in Ghana, where the use of mobile phones to contact patients was compared with home visits to identify AEs related to artemisinin-based combination therapy for treating uncomplicated malaria found a slightly higher reporting rate with mobile phones, and that it was possible to interview the majority of patients within a few days [17]. In Uganda, an SMS-based reporting system for monitoring malaria diagnosis and treatment improved timeliness in data reporting [18]. A pilot study in rural districts of Kenya using mobile text messaging for malaria surveillance demonstrated that phone reporting can improve timeliness and reporting of data [19]. Semi-structured interviews and focus group discussions conducted in conjunction with this study indicated that active surveillance was appreciated by community members, but CHWs found this time-consuming and considered enhanced spontaneous reporting to be effective; however, technical difficulties in terms of electricity supply and internet connections were noted. The authors intend to publish results from this aspect of the study separately.

## Conclusions

This study has shown that involving CHWs in safety reporting, and training nurses and CHWs to report using a mobile phone application, can be used to enhance safety reporting and improve timeliness of notifications, but the approach relies on training and supervision of CHWs and health facility staff, effective community sensitization, and a central team to process reports and provide feedback. These strategies could be adopted in sentinel sites when new public health programs are introduced and scaled up. Enhanced safety monitoring using this approach could be established in SMC areas; however, for comparison, laboratory investigation should be strengthened and baseline incidence should be collected prior to SMC campaigns.

## Electronic supplementary material

Below is the link to the electronic supplementary material.

Supplementary material 1 (DOCX 504 kb)
