# Supplementary material for: Evaluation of Two Strategies for Community-Based Safety Monitoring during Seasonal Malaria Chemoprevention Campaigns in Senegal, Compared with the National Spontaneous Reporting System
Source: Pharmaceut Med. 2018 Jun 1;32(3):189–200. doi: 10.1007/s40290-018-0232-z (PMC6006231; doi:10.1007/s40290-018-0232-z)
Supplement: Supplementary file 1 — Supplementary material 1 (DOCX 504 kb) [file 40290_2018_232_MOESM1_ESM.docx]

**Supplementary information**

**Evaluation of two strategies for community-based safety monitoring during Seasonal Malaria Chemoprevention campaigns in Senegal, compared with the national spontaneous reporting system**

Running title: Community-based safety monitoring during Seasonal Malaria Chemoprevention campaigns in Senegal

Jean-Louis A. Ndiaye, Ibrahima Diallo, Youssoupha NDiaye, Ekoue Kouevidjin, Ibrahima Aw, Fassiatou Tairou, Tidiane Ndoye, Christine M. Halleux, Isaac Manga, Mbaye Niang Dieme, Medoune Ndiop, Babacar Faye, Piero Olliaro, Corinne S. Merle, Oumar Gaye, Paul Milligan

Email addresses:

Jean-Louis A. Ndiaye*, [jeanloab.ndiaye@ucad.edu.sn](mailto:jeanloab.ndiaye@ucad.edu.sn), tel : +2216445917

Ibrahima Diallo, [haril76@yahoo.fr](mailto:haril76@yahoo.fr)

Yousoupha NDiaye, [youlebou@gmail.com](mailto:youlebou@gmail.com)

Ekoue Kouevidjin, [ekoue.kouevidjin@gmail.com](mailto:ekoue.kouevidjin@gmail.com)

Ibrahima Aw, [ibouaw80@gmail.com](mailto:ibouaw80@gmail.com)

Fassiatou Tairou, [fassiatht@yahoo.fr](mailto:fassiatht@yahoo.fr)

Tidiane Ndoye, tndoye16@gmail.com

Christine M. Halleux, [halleuxc@who.int](mailto:halleuxc@who.int)

Isaac A. Manga, [akhenmanga@yahoo.fr](mailto:akhenmanga@yahoo.fr)

Mbaye Niang Dieme, [mbndieme@hotmail.com](mailto:mbndieme@hotmail.com)

Medoune Ndiop, [mnzop5@gmail.com](mailto:mnzop5@gmail.com)

Babacar Faye, [bfaye67@yahoo.fr](mailto:bfaye67@yahoo.fr)

Piero Olliaro, [olliarop@who.int](mailto:olliarop@who.int)

Corinne S. Merle, [merlec@who.int](mailto:merlec@who.int)

Oumar Gaye, [oumar.gaye@ucad.edu.sn](mailto:oumar.gaye@ucad.edu.sn)

Paul J. Milligan, [Paul.Milligan@lshtm.ac.uk](mailto:Paul.Milligan@lshtm.ac.uk), ORCID 0000-0003-3430-3395

Figure S1 Map of the study area

Figure S2 Incidence of reports of vomiting by age, in children 0-10 years of age, after SMC distributions in September, October and November, observed through passive and active methods of surveillance.

Figure S3 Number of reports of adverse events, by day since start of SMC treatment, in the Enhanced Spontaneous Reporting areas (A), and the areas with Active Surveillance (B).

Table S1: Number of adverse event reports (with any symptom), in each health post.

Table S2: Number of symptoms reported per case.

Table S3: Association between symptoms: Odds ratios for the associations between symptom pairs.

Table S4 Number of times each symptom was reported.

Table S5: SWPO for the Active surveillance.

Table S6: SWPO for the enhanced spontaneous reporting involving CHWs and using mobile phones.

Table S7: Classification of the association of adverse events with drug intake.

Figure S1: Map of the study area


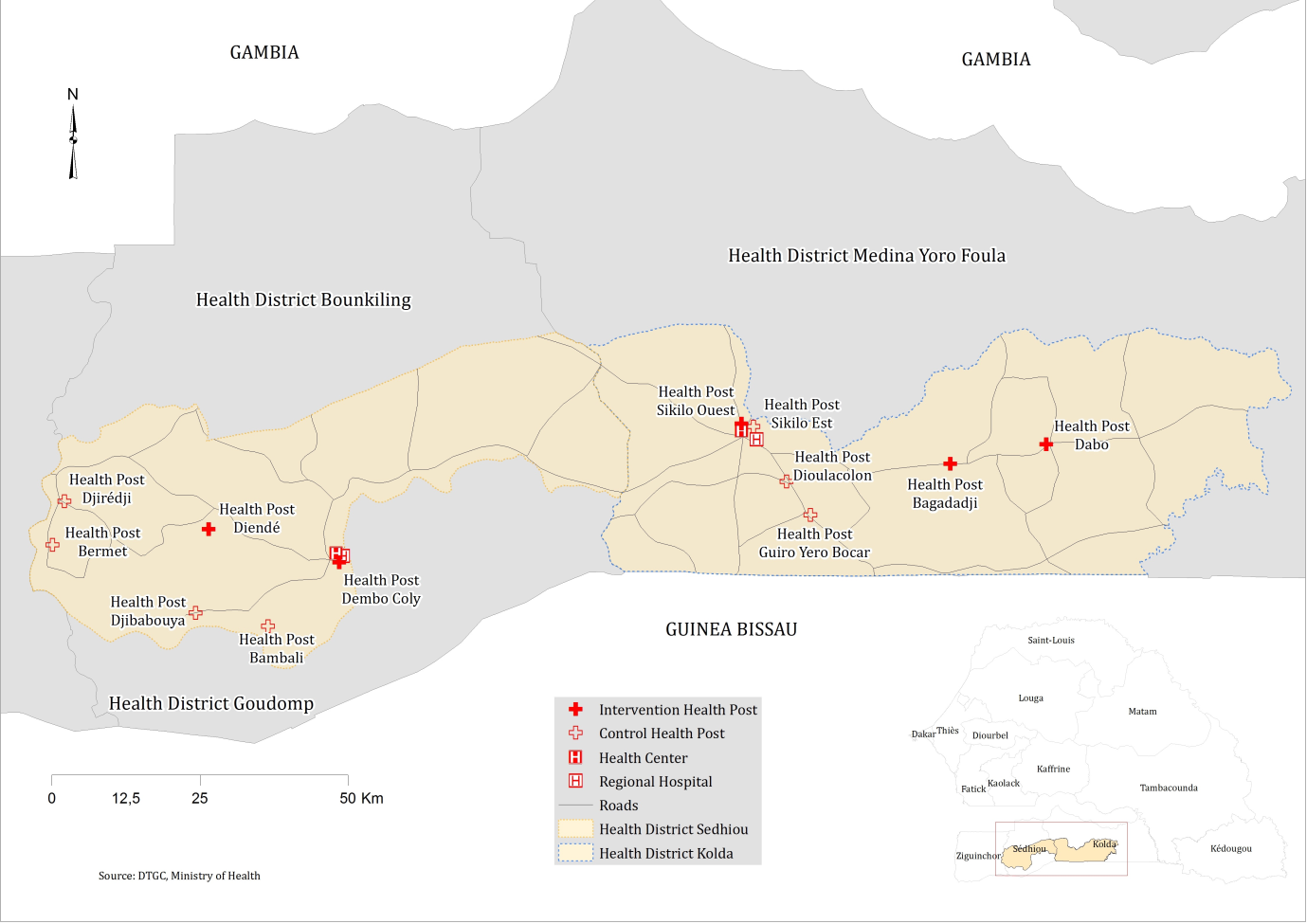


Figure S2: Incidence of reports of vomiting by age, in children 0-10 years of age, after SMC distributions in September, October and November, observed through passive and active methods of surveillance.

Figure S3: Number of reports of adverse events, by day since start of SMC treatment, in the Enhanced Spontaneous Reporting areas (A), and the areas with Active Surveillance (B).

1. Enhanced spontaneous reporting

1. Active surveillance: (day symptom started – passive is when symptoms lead to care seeking hence distribution shifted)

| District | PV method | Poste | Sep | Oct | Nov |
| --- | --- | --- | --- | --- | --- |
| Sedhiou | **Enhanced spontaneous** | **Diende** | 244 (51) | 238 (49) | 83 (16) |
|  | **reporting** | **Dembo Coly** | 326 (43) | 168 (22) | 86 (11) |
|  | **National system** | **Djibabouya** | 17 (5.9) | 5 (1.7) | 7 (2.3) |
|  |  | **Bambaly** | 8 (2.1) | 4 (1.0) | 2 (0.4) |
|  |  | **Djiredji** | 6 (1.5) | 2 (0.5) | 4 (1.0) |
| Kolda | **Active** | **Bagadadji** | 60 (14) | 68 (15) | 27 (5.9) |
|  | **surveillance** | **Dabo** | 41 (13) | 91 (30) | 52 (17) |
|  |  | **Sikilo Ouest** | 205 (75) | 106 (35) | 30 (9.6) |
|  | **National system** | **Sikilo Est** | 54 (19) | 19 (7.2) | 7 (2.6) |
|  |  | **Dioulacolon** | 2 (0.4) | 0 (0.0) | 0 (0.0) |
|  |  | **Guiro Yero Boucar** | 21 (7.4) | 0 (0.0) | 0 (0.0) |
|  |  | **All** | 984 (22.5) | 701 (15.7) | 298 (6.5) |

Table S1: Number of adverse event reports (with any symptom), in each health post (rate/1000):

Table S2: Number of symptoms reported per case:

|  | No. of symptoms: | |  |  |  |  |  |
| --- | --- | --- | --- | --- | --- | --- | --- |
| Surveillance method | 1 | 2 | 3 | 4 | 5 | Total | Mean |
| National system | 51% (80) | 36% (57) | 11% (17) | 2.5% (4) | 0% (0) | 158 | 1.65 |
| Enhanced spontaneous reporting | 50% (568) | 36% (413) | 13% (145) | 1.6% (18) | 9% (1) | 1145 | 1.66 |
| Active surveillance | 79% (535) | 21% (142) | 0.4% (3) | 0% (0) | 0% (0) | 680 | 1.22 |

Table S3: Association between symptoms: Odds ratios for the associations between symptom pairs.

|  |  | Control | Enhanced  spontaneous  reporting | | Active  surveillance | |  |
| --- | --- | --- | --- | --- | --- | --- | --- |
| Symptom 1 | **Symptom 2** | **Odds ratios symptom1:symptom2** | | | | | P-value from test of homogeneity |
| Vomitting | **Abdominal pain** | 0.74 | 0.46 | 0.35 | | 0.119 | |
| Vomitting | **Diarrhoea** | 0.26 | 0.80 | 0.27 | | <0.001 | |
| Vomitting | **Fever** | 0.20 | 0.80 | 0.17 | | <0.001 | |
| Abdominal pain | **Diarrhoea** | 0.59 | 0.30 | 0.13 | | 0.1461 | |
| Abdominal pain | **Fever** | 1.06 | 0.43 | 0.14 | | <0.001 | |
| Diarrhoea | **Fever** | 0.27 | 0.57 | 0.10 | | 0.0043 | |

Table S4 Number of times each symptom was reported:

|  | Sedhiou | | Kolda | |  |
| --- | --- | --- | --- | --- | --- |
| Symptom | Enhanced spontaneous reporting | National system | Active surveillance | National system | Total |
| Fever | 581 | 7 | 201 | 28 | 817 |
| Vomitting/nausea | 389 | 34 | 335 | 47 | 805 |
| Abdominal pain | 312 | 17 | 174 | 49 | 552 |
| Diarrhoea | 277 | 16 | 82 | 10 | 385 |
| Headache | 146 | 5 | 15 | 21 | 187 |
| Cough | 68 | 1 | 1 | 10 | 80 |
| Weakness | 47 | 1 | 6 | 2 | 56 |
| Loss of appetite | 31 | 0 | 1 | 0 | 32 |
| Dizziness | 17 | 0 | 3 | 0 | 20 |
| Rash | 10 | 0 | 7 | 2 | 19 |
| Chills | 0 | 0 | 0 | 10 | 10 |
| Red eyes | 8 | 0 | 0 | 0 | 8 |
| Lethargy | 6 | 0 | 1 | 0 | 7 |
| Itching | 3 | 0 | 2 | 0 | 5 |
| Cold | 3 | 0 | 0 | 0 | 3 |
| Bloody stools | 2 | 0 | 0 | 0 | 2 |
| Abcess | 1 | 0 | 0 | 0 | 1 |
| Swollen face | 1 | 0 | 0 | 0 | 1 |
| Runny nose | 1 | 0 | 0 | 0 | 1 |
| Haemoptysis | 1 | 0 | 0 | 0 | 1 |
| Chest pain | 1 | 0 | 0 | 0 | 1 |
| Crying | 1 | 0 | 0 | 0 | 1 |
| Muscle pain | 0 | 0 | 0 | 1 | 1 |

Table S5: SWPO for the Active surveillance:

| **Successes** | **Weaknesses** |
| --- | --- |
| Community aware of pharmacovigilance  Active follow-up detects adverse events that would not otherwise be reported  Involvement of the “comité de santé” and health district team  Involvement of community leaders  Fast and effective management of AE cases  Strengthening the existing PV system | Delay in transmission of AE notification forms  Difficulty related to the filling of the forms  Risk of loss of the notification forms  Work overload of the community health workers |
| **Potentialities** | **Obstacles** |
| Involvement of community organizations (e.g. comité de veille et d’alerte communautaire) in the sensitization of the population and the follow-up of AEs  Involvement of young people in the sensitization of the population and the follow-up of AEs  Set-up of a monitoring and warning committee (parents, CHWs) to report AEs and send patients to the health centre | Lack of coordination between actors involved at community and central level  No feedback at community level after identification and notification of cases  Stock out of drugs to treat AEs  Lack of logistics to follow up AEs |

Table S6: SWPO for the enhanced spontaneous reporting involving CHWs and using mobile phones

| **Successes** | **Weaknesses** |
| --- | --- |
| Strengthening of AE notification system  Improved reporting of cases (detection and documentation)  Prompt transmission of information about the cases  Availability of the AEs database promptly  Early warning on cases at central level  Coordination between health staff, CHWs  Involvement of community members (DSDOM)  Awareness: interpersonal communication  Monitoring at Community level: improving the spontaneous reporting  Increase AE reporting at health posts level  Management and control of AE cases at community and central levels | Difficult to use the touch phones  DSDOM work overload (drug delivery and AE monitoring)  Difficulty to have a normal telephone network to download the notification form and to send the form  Network problem, sends multiple/duplicate notifications |
| **Potentialities** | **Obstacles** |
| Collaboration between ministry of health team and academics experts  Involvement of community stakeholders (e.g. groupement des femmes, ) in the sensitization  Availability of community human resources (DSDOM) | Lack of adequate logistics: phone card and in case of rain  Risk of lightning during the rainy season with phone use.  Problem with internet network in the study area  Lack of electricity: Problem to recharge mobile phone |

Table S7: Classification of the association of adverse events with drug intake:

| Certain | - Event or laboratory test abnormality, with plausible time relationship to drug intake - Cannot be explained by disease or other drugs - Response to withdrawal plausible (pharmacologically, pathologically) - Event definitive pharmacologically or phenomenologically (i.e. an objective and specific medical disorder or a recognized pharmacological phenomenon) - Rechallenge satisfactory, if necessary |
| --- | --- |
| Probably/likely | - Event or laboratory test abnormality, with reasonable time relationship to drug intake - Unlikely to be attributed to disease or other drugs - Response to withdrawal clinically reasonable - Rechallenge not required |
| Possible | - Event or laboratory test abnormality, with reasonable time relationship to drug intake - Could also be explained by disease or other drugs - Information on drug withdrawal may be lacking or unclear |
| Unlikely | - Event or laboratory test abnormality, with a time to drug intake that makes a relationship improbable (but not impossible) - Disease or other drugs provide plausible explanations |
| Conditional/  Unclassified | - Event or laboratory test abnormality - More data for proper assessment needed, or - Additional data under examination |
| Unassessable/  Unclassifiable | - Report suggesting an adverse reaction - Cannot be judged because information is insufficient or contradictory - Data cannot be supplemented or verified |
